# Imaging Platelet Processes and Function—Current and Emerging Approaches for Imaging *in vitro* and *in vivo*

**DOI:** 10.3389/fimmu.2020.00078

**Published:** 2020-01-31

**Authors:** Samantha J. Montague, Yean J. Lim, Woei M. Lee, Elizabeth E. Gardiner

**Affiliations:** ^1^ACRF Department of Cancer Biology and Therapeutics, The John Curtin School of Medical Research, The Australian National University, Canberra, ACT, Australia; ^2^Research School of Electrical, Energy and Materials Engineering, The Australian National University, Canberra, ACT, Australia

**Keywords:** platelet, thrombosis, microscopy-brightfield, polarized light, interference, scanning electron, microscope, receptors

## Abstract

Platelets are small anucleate cells that are essential for many biological processes including hemostasis, thrombosis, inflammation, innate immunity, tumor metastasis, and wound healing. Platelets circulate in the blood and in order to perform all of their biological roles, platelets must be able to arrest their movement at an appropriate site and time. Our knowledge of how platelets achieve this has expanded as our ability to visualize and quantify discreet platelet events has improved. Platelets are exquisitely sensitive to changes in blood flow parameters and so the visualization of rapid intricate platelet processes under conditions found in flowing blood provides a substantial challenge to the platelet imaging field. The platelet's size (~2 μm), rapid activation (milliseconds), and unsuitability for genetic manipulation, means that appropriate imaging tools are limited. However, with the application of modern imaging systems to study platelet function, our understanding of molecular events mediating platelet adhesion from a single-cell perspective, to platelet recruitment and activation, leading to thrombus (clot) formation has expanded dramatically. This review will discuss current platelet imaging techniques *in vitro* and *in vivo*, describing how the advancements in imaging have helped answer/expand on platelet biology with a particular focus on hemostasis. We will focus on platelet aggregation and thrombus formation, and how platelet imaging has enhanced our understanding of key events, highlighting the knowledge gained through the application of imaging modalities to experimental models *in vitro* and *in vivo*. Furthermore, we will review the limitations of current imaging techniques, and questions in thrombosis research that remain to be addressed. Finally, we will speculate how the same imaging advancements might be applied to the imaging of other vascular cell biological functions and visualization of dynamic cell-cell interactions.

## Introduction

### Imaging and Platelets

Platelets are minute disk-shaped cells that are produced from megakaryocytes and have prominent roles in hemostasis. Platelets contain many granules that hold growth factors, chemokines, and other platelet-activating molecules and proteins and have an open canalicular system (OCS) important for protein transport. Furthermore, platelets also have a plethora of membrane surface receptors that are vital for platelet activation and thus, function, and for interactions with other immune cells including leukocytes (1, 2), malaria-infected red cells (3) and adaptive immune cells (4–6).

Excellent historical accounts of the first visual observations of platelets have been extensively reviewed (7, 8). Notable observations were made by Max Shultze (9) and Bizzozero (10), both pioneers of cell biology who adapted existing oil immersion microscopes within moist chambers to visualize blood “particles” and describe them as another blood component distinct from leukocytes and erythrocytes. Bizzozero also described how platelets had a physiological role in stopping hemorrhages (bleeds) in vessels (10).

Since the development of rudimentary immersion lenses, a number of improved and unique optics and laser technologies have emerged. These modern imaging tools are designed to observe diverse molecular and morphological changes of cells and/or dynamic interactions within a network of living cells *in vitro* and *in vivo*. For analyses of blood cell function, applications have largely focused on immune and red blood cell (RBC) biology as these cells are >5 μm in diameter and are well-suited for most commercial micro-imaging tools, where imaging in three dimensions at high spatial resolution is achievable.

Platelets, on the other hand, have received less focus, due to both being small in size (around 2–3 μm) and with the potential to be rapidly activated. The biophysical properties of platelets are distinct in their sensitivity to changes in blood fluid shear force, thus capturing platelet events at physiological flow rates requires high performance imaging systems as platelets are highly susceptible to motion blurring. Thus, platelet imaging under physiological flow conditions tests the limitation of spatial-temporal imaging resolution where sub-platelet structures are not visible (11).

### Platelets, the Infantry of the Blood

Platelets circulate in the blood in a resting, quiescent state, with circulating levels maintained at a constant level within the normal range 150–400 × 10^9^ platelets per liter of blood in healthy people (12). Human platelets circulate for between 7 and 10 days and are selectively removed by resident cells of the liver or spleen for clearance unless they are consumed as part of a hemostatic response (13). Although platelets do not contain a nucleus (therefore no DNA), they contain RNA, ribosomes, mitochondria, and a number of storage organelles and granules, which are dynamically regulated during normal platelet function (14, 15).

### Granules

Platelet alpha, dense, gamma, and lambda granules contain chemokines (platelet factor 4; PF4, CXCL7), growth factors (vascular endothelial growth factor; VEGF, platelet-derived growth factor; PDGF), coagulation proteins and platelet-activating molecules (ADP, Factor V, Factor XIII, fibrinogen, and von Willebrand Factor; VWF) as well as lysosomes/proteolytic enzymes (16–18). Release of platelet granular contents helps stabilize platelet aggregates, enhance further platelet recruitment, and amplifying wound repair and immunological and inflammatory processes (15, 19–21). Often these granule contents can be used to indicate and quantify platelet activation.

### Adheso-Signaling Receptors

Platelets have a host of membrane-associated receptors that engage with one or more counter-receptors or plasma/extracellular matrix proteins. Of major importance, glycoprotein (GP) Ib-IX-V, which binds VWF as well as P-selectin, Factors XI and XII, leukocyte integrin αMβ2, collagen, thrombin and kininogen, and GPVI, which binds collagen, fibrin and laminin (22), initiate platelet adhesion events. These receptors act in concert (23) to translate cues from the surrounding vascular environment to mediate molecular signaling pathways that lead to platelet activation, platelet adhesion as well as mediating interactions with other cells (24, 25). The goal is for platelets to adhere and seal the damaged vessel area, thus maintaining hemostasis (26). Platelet receptor engagement triggers phosphorylation and activation of intracellular molecules (Src family kinases, phosphoinositol-3 kinase and protein kinase C), degranulation, and the rearrangement of the cytoskeleton causing platelet shape change (27). Ultimately, these activation steps result in the activation of the platelet-specific integrin αIIbβ3, which non-covalently binds dimeric plasma fibrinogen as well as potentially other plasma proteins (fibronectin, cadherins, VWF) thus bridging adjacent platelets (28).

As platelets also contain an OCS, receptor engagement and cytoskeletal rearrangement coordinates the exposure of this specialized internal membrane network that is important for protein transport (29) and amplification of prothrombotic responses. The cytoskeletal rearrangement enables platelet receptors to cluster (30, 31) which amplifies signaling events and helps stabilize platelet contact points. Activated platelet membranes become negatively charged through the exposure of phosphatidylserine and this mediates procoagulant (thrombin generating) capacity (32). Phosphatidylserine exposure can also occur in pathophysiological settings such as on exposure of murine platelets to antiplatelet autoantibodies (33). An additional consequence of receptor activation is the metalloproteolytic shedding of the ligand-binding ectodomains of GPIbα (the ligand-binding portion of GPIb-IX-V) and GPVI receptors. Through this metalloproteolytic process, thrombus propagation may be controlled and limited (34, 35).

### The Process of Thrombus Formation

Thrombosis is an exaggerated and generally undesired form of hemostasis where there is uncontrolled platelet adhesion and aggregation, leading to increased thrombin formation, and fibrin generation (36). Large thrombi (blood clots) may occlude blood vessels or undergo embolization, where the thrombi break apart and pieces move to occlude smaller vessels causing strokes and myocardial infarction (37). Arterial thrombosis usually is triggered by rupture of a collagen- and tissue factor-rich stenotic plaque at relatively high (1,000–5,000 s^−1^) wall shear rates, which are sufficient to unfold VWF and activate platelets. Venous thrombosis occurs at very low or static (0–200 s^−1^) shear rates with contributions from the vascular bed and inflammatory cells (38). Together, acute venous and arterial thrombosis accounts for the most common causes of death in developed countries (39–41).

**Figure 1:**
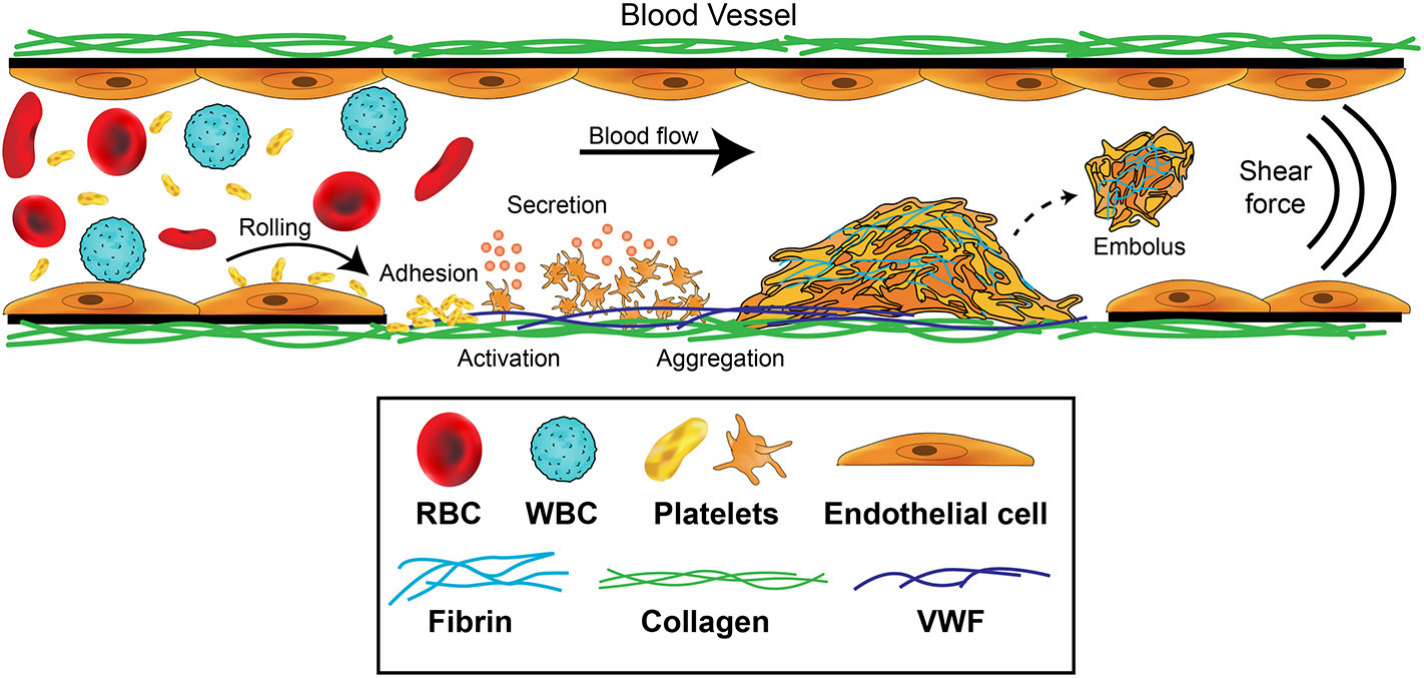
Platelet contributions to thrombus formation. Platelets circulate in the blood stream in a quiescent (resting) state. When exposed extracellular matrix proteins such as von Willebrand Factor (VWF) or collagen are detected at the site of injury, platelets are induced to roll, and then adhere. The GPIb-IX-V complex and GPVI receptors on platelets orchestrate this adhesion and activation process. Adherent platelets become activated, expose P-selectin and phosphatidylserine, and secrete secondary mediators such as ADP and thromboxane. This promotes platelet recruitment and activation of αIIbβ3 which mediates platelet aggregation by binding plasma fibrinogen. Coagulation is also activated resulting in fibrin formation following thrombin cleavage of fibrinogen, leading to the consolidation of the platelet aggregate into a thrombus and healing of the damaged area. Fibrinolytic processes eventually dissolve the formed thrombus, causing the thrombus to embolize. Thrombosis occurs when there is increased coagulation and exaggerated thrombus formation and/or reduction of fibrinolytic processes, potentially leading to occlusion of the blood vessel.

Platelet aggregation leading to thrombus formation is a multistep adhesion process (Figure 1) involving distinct receptors and adhesive ligands, with the contribution of individual receptor-ligand interactions to the aggregation process dependent on the prevailing blood flow conditions (42, 43). Platelets normally circulate in a quiescent, latent form but initially roll, then adhere at sites of endothelial injury, where matrix proteins such as collagen, VWF or laminin are exposed. If the rheological conditions are altered such that non-laminar disrupted flow is present, platelet activation is also immediately triggered (36, 44, 45). Engagement of GPIbα by the A1 domain of VWF under a shear force is critical for generation of ligand–receptor signals (46, 47). Intracellular signals then trigger platelets to change shape and flatten, cluster receptors, undergo calcium flux, generate reactive oxygen species and begin to degranulate. These steps serve to stabilize the adherent platelet, amplify the platelet activation, and enhance recruitment of additional platelets to the aggregate. Platelets are able to form stable adhesion contacts at all shear rates found throughout the vasculature (48) and activation can occur directly in flowing blood—within regions of a flowing blood column that can impart either intermittent or sustained elevated shear exposure in the absence of blood vessel wall contact (49).

Development of high-speed imaging approaches have enabled many laboratories to evaluate and quantify this process *in vitro* and our understanding of how receptors, vascular constituents, rheology and secondary messengers released from platelets contribute to this process has expanded. Nonetheless, important additional contributions of RBCs and leukocytes as well as contributions from specific vascular beds, coagulation processes and blood rheology considerations are generally missing from experiments *in vitro*, meaning that many aspects of this system remain to be well-defined.

### Imaging Platelet Function *in vitro* to Advance Our Understanding of Thrombosis

In the modern era, platelet function can be readily imaged *in vitro* using advanced light-based microscopy systems with phase contrast or fluorescence capabilities (Table 1). In many cases, the isolation of human platelets from anticoagulated blood is desirable to reduce cellular autofluorescence (68) and allow clearer visualization of platelets. Platelet isolation is rapidly achieved using low speed centrifugation (110 *g*), to obtain a preparation of platelet-rich plasma (PRP; platelets plus all plasma proteins) with minimal numbers of RBCs and leukocytes. Removal of microparticles can be achieved by ultracentrifugation of isolated plasma at >100,000 *g* and used for platelet resuspension. Using selected anticoagulants and wash buffers that control pH well, plasma proteins can be “washed” away from platelets to generate a washed platelet preparation that is free of all plasma components. This fractionation and preparation is ideal for single platelet imaging and spreading. In summary, the single cell imaging techniques have utility to examine specific surface receptors, platelet cytoskeletal changes, interactions with immobilized ligands such as collagen and fibrinogen, or platelet-cell interactions. Washed platelets, PRP and anticoagulated whole blood can be also used in microfluidic-based systems to examine thrombus formation under conditions found in flowing blood.

**Table 1:** Imaging techniques and applications for platelet research *in vitro*.

**Imaging method**	**Key points**	**Resolution**	**Platelet/cell imaging applications**	**Limitations**	**References**
Conventional/ Bright-field/Widefield	Uses visible light or high intensity light sources to illuminate a sample	*L* = 200–300 nm *Ax* = 500–800 nm	Thrombus formation >Microfluidics Large platelet aggregates	Low resolution Not suitable for single cell evaluation Limited by wavelength of light and NA of objective lens	(50)
Confocal/CLSM	Uses light to illuminate a sample through a pinhole to improve optical resolution Uses spatial filtering to block out-of-focus light	L = >200 nm (reflection) >250 nm (fluorescence)	Thrombus formation Platelet spreading >Surface receptor information Healthy controls vs. patients differences > wild type mice vs. knock out mice differences	Fluorescence label Surface area and receptors data Minimal information on cytoskeleton	(50–52)
QPM/DHM	Generates quantitative measurements from shifts in phase	L = >270 nm	Volumetric measurements of thrombus formation	No receptor profile details Requires complex post-image analysis	(53–56)
CLEM/3D cryoEM	Approaching atomic level analysis of ultrastructural changes, adhesion, and granule secretion	L = < 1 nm	Platelet secretion; Megakaryocyte positioning in sinusoids and platelet production (applied in intravital setting)	Samples need to be mounted on a grid; precise solvent requirements	(57–60)
STED	Confocal excitation beam overlaid by a depletion beam to inhibit fluorescence emission at target area of interest	L = 50–60 nm	Platelet protein distribution when co-incubated with cancer cells Platelet protein storage	Deconvolution required Need specific STED dyes Decreased scan step size + increased acquisition time	(61–64)
SMLM SIM PALM (d) STORM PAINT	Illumination that relies on single molecule switching by stochastic excitation Switching on/off of a fluorescent molecule or through excitation	L = >20 nm *Ax* = >50 nm	Platelet cytoskeleton proteins Actin nodules/tubulin Megakaryocyte structure and function Synapses Platelet receptor co-localization and receptor clustering	Computer power/software and storage Vast number of data points Post-data analysis and complex image reconstruction Specific photoswitchable and activatable fluorescence labels	(31, 65–67)

*A non-exhaustive list of imaging techniques used to study platelet spreading, function, receptor profiles, and platelet protein/cytoskeletal protein organization in vitro. Rows highlighted in blue are examples of microscopy approaches that operate at nanoscopic/super resolution limits of diffraction. L, Laterally; Ax, Axially; NA, numerical aperture; CLSM, Confocal Laser Scanning Microscopy; QPM, Quantitative Phase Microscopy; DHM, Digital Holographic Microscopy; CLEM, Correlative light-electron microscopy; cryoEM, Cryogenic Electron Microscopy; SPIM, Selective Plane Illumination Microscopy; STED, Stimulated Emission Depletion; SMLM, Single-Molecule Localization Microscopy; SIM, Structured Illumination Microscopy; PALM, Photo-Activated Localization Microscopy; STORM, Stochastic Optical Reconstruction Microscopy; PAINT, Point Accumulation for Imaging Nanoscale Topography*.

### Micro-Imaging Platelets in a Dish

Platelets can quickly change from a rounded, non-adherent form to adhere and undergo rapid shape change (flatten) when exposed to purified immobilized ligands such as extracellular matrix proteins collagen and laminin and adhesive proteins, including fibrinogen and VWF. This “spreading” effect can lead to the formation of filopodia and lamellipodia with subsequent ability to actively mobilize, which requires cytoskeletal protein rearrangement, including polymerization of actin, and other cytoskeletal proteins (69). Platelet adhesion and extent of spreading (area covered) can be quantified.

**Figure 2:**
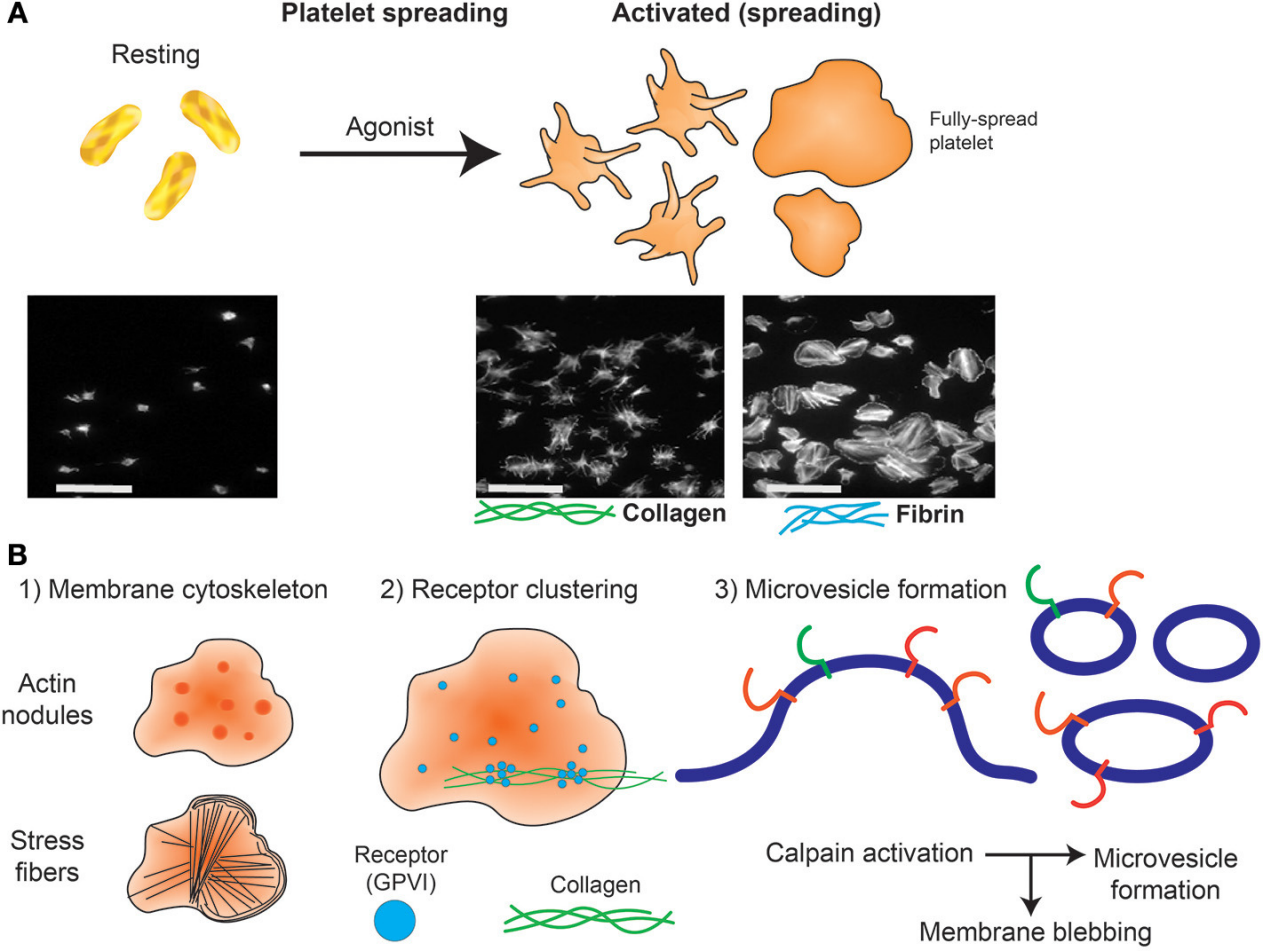
Platelet spreading. **(A)** Under resting conditions platelets normally are non-adherent. Upon exposure to an activating agonist, platelets change shape by reorganizing cytoskeletal elements, leading to the formation of filopodia, followed by lamellipodia and an increase in surface area. When platelets are exposed to immobilized ligands in experiments *in vitro*, this shape change is known as platelet spreading. Light microscopy images shows actin arrangement and morphology of phalloidin-treated platelets exposed to non-coated coverslips (left) or coverslips pre-coated with collagen (middle) or fibrin (right). Images were taken using an inverted bright-field fluorescence microscope. Scale bar = 20 μm. **(B)** Schematic of processes that can be imaged during platelet spreading *in vitro* include (1) Cytoskeletal protein rearrangement, such as formation of actin nodules, microtubule organization and generation of stress fibers; (2) super resolution microscopy (dSTORM, SIM) can capture GPVI clustering (purple dots) and alignment along collagen fibers (green lines); (3) microvesicle formation can be imaged using optical systems that provide resolution below 150 nm; discrete cytoskeletal rearrangement occurs alongside calpain-dependent processes, where calcium-sensitive proteases detach membrane proteins, allowing membrane blebbing required for microvesicle release from platelets and megakaryocytes.

Widefield microscopy, including phase contrast, total internal reflection fluorescence (TIRF) (70, 71), reflectance interference contrast (RIC) (72), differential interference contrast (DIC) (73) and confocal microscopy with fluorescence capabilities (74) have enabled visualization of activation events in real time including clustering of platelet receptors and cytoskeleton rearrangement following platelet contact with immobilized ligands. Washed platelets are usually resuspended in permeabilizing buffer containing fluorescently-tagged antibodies or probes targeting actin and tubulin (51, 75, 76), and allowed to spread at 37°C for 30–120 min on microscope slides coated with an immobilized ligand to visualize changes in cytoskeletal rearrangement (Figure 2B). Actin-mediated cytoskeletal rearrangements allow the formation of filopodia and then lamellipodia and microscopy has identified differential contributions of each of these structures to a forming thrombus (77). Live cell imaging has demonstrated roles for GTPase proteins including Cdc24, RAC1, and RhoA (78). Widefield microscopy has also been valuable in assessing megakaryocyte (platelet parental cell) function, including studying roles of receptors, cytokines and growth factors in pro-platelet formation (79–81).

Widefield microscopy imaging together with the availability of genetic data has helped identify and characterize platelet defects in patients with syndromes including Scott syndrome (82), Wiskott-Aldrich syndrome (65), and Filamin A disorders (83, 84). These syndromes are challenging to detect or evaluate using conventional platelet function testing due to associated thrombocytopenia (low platelet count). Of note, platelet spreading assays, which are not affected by low platelet count, can help define bleeding phenotypes in patient samples that are negative for an aggregation defect (85). The combination of biological optical microimaging with genomic information has opened up new avenues to test and evaluate these rare conditions that are not limited by low platelet counts but are still constrained by the limits of optical diffraction (86, 87).

### Nanoscale Imaging of Single Platelets

Initial ultra-high resolution imaging studies of the platelet cytoskeleton and membrane glycoproteins were assessed using electron microscopy (EM) (88–90). EM is a highly specialized and time-consuming technique that provides excellently detailed nanometer scale level imaging resolution of platelet ultrastructure including intracellular organelles, cytoskeletal components, and storage granules that is beyond the resolution limits of conventional light microscopy. EM has been used to describe platelet dysfunction disorders, such as Gray Platelet syndrome, the rare congenital autosomal recessive bleeding disorder caused by an absence or deficiency in alpha granules (91, 92).

Scanning and transmission EM protocols generally require multiple washing of small portions of sample and can also integrate immunolabeling and negative staining techniques. Transmission EM requires thin tissue sections through which electrons can pass generating a projection image of the interior of cells, structure and organization of protein molecules and cytoskeletal filaments, and the arrangement of protein in cell membranes (by freeze-fracture). Scanning EM provides a wealth of information about surface topography, atomic composition and distribution of immunolabels. A limitation of EM samples obtained from platelets from patients and thrombi are they often become unviable at the time of processing and this imposes limitations on the types of biological questions that can be pursued.

The emergence of super resolution microscopy and other nanoscopy techniques (93–95) have overcome several limitations of traditional light-based approaches to achieve nanometer resolution. Unlike EM techniques, these samples can be prepared using regular biochemical processes that preserve biological functions. Amongst many nanoscopic techniques, Structured Illumination Microscopy (SIM) and Single Molecule Localization Microscopy (SMLM) approaches (61) have provided unique insights into cytoskeletal protein clusters of actin nodules (65), tubulin and actin stress fibers (96, 97), and cytoskeletal rearrangement during platelet activation (98, 99). These approaches also have greatly improved knowledge of surface receptor co-localizations. For example, the platelet receptor GPVI has been shown to dimerize and cluster along collagen fibers (Figure 2) and is co-localized with integrin α2β1 (31). It will be interesting to apply these high resolution techniques to determine whether other receptor-ligand interactions, such as GPIb-IX-V on immobilized VWF, also demonstrate dynamic movement, and whether GPVI clusters in this way on fibrin(ogen) or other immobilized GPVI ligands. The consequences of receptor clustering on platelet aggregate formation and stability, and whether clustered receptors are protected from proteolytic cleavage by metalloproteinases such as A Disintegrin And Metalloproteinase (ADAM) 10 (100) are additional research questions that can now be addressed.

SIM imaging approaches are well-suited to evaluate platelet-specific defects in individuals and or genetically modified animals with congenital deficiencies in protein expression. For example, SIM has been applied to study spreading behavior and changes in cytoskeletal rearrangement in platelets with cytoskeletal protein deficiency; such as ARPC1-deficiency, where reduced actin-related protein 2/3 complex (Arp2/3) led to aberrant platelet spreading (66) and Wiskott-Aldrich syndrome protein (WASp) deficient platelets from patients and WASp knockout mice resulted in reduced actin nodule formation (65). With the expanding implementation of SIM in other microscopy methods (e.g., TIRF microscopy), we anticipate SIM to have increased applications in high to super-resolution imaging of platelet and thrombus behavior (101).

High resolution microscopy has also been valuable in assessing megakaryocyte (platelet parental cell) function, including studying roles of receptors, cytokines, and growth factors in pro-platelet formation (57, 80, 81). However, these imaging techniques have been developed for a static system, which does not permit implementation of fluid shear stress, a crucial physiological driver of platelet production. The next frontier, therefore, is to couple these imaging processes to microfluidic systems, and examine platelet and megakaryocyte processes under conditions found in flowing blood.

Brown et al. used electron tomography coupled with intravital correlative light-electron microscopy (CLEM) to capture thrombopoiesis events in real time and calculate megakaryocyte membrane parameters during this process of platelet production. They identified that mass fusion between internal and external membranes allows megakaryocytes to extend multiple protrusions rather than proplatelets into the marrow sinusoidal vessel space (58).

## Microfluidics Imaging of Platelets: Recapitulating Thrombus Formation *in vitro*

### Laboratory Research and Extending to the Clinical Sector

Microfluidic devices and flow-based systems provide good avenues to study these concepts in combination, thus allowing coagulation, platelet function, and roles of shear to be studied simultaneously, using small quantities of blood (Table 1). Simple single channel microfluidic systems generally uses glass capillary tubes (optically clear) or conduits made using a mask to produce channels usually of 50–100 μm thick channels in polydimethylsiloxane (PDMS) which are mounted on glass coverslips (102). The channels or capillaries are coated with an adhesive ligand (103, 104). A syringe pump either pulls or pushes antibody- or fluorescently labeled blood, PRP or washed platelets in the presence of anticoagulant (generally trisodium citrate and PPACK), through the channel at constant shear rates which are determined by the velocity of the flow and viscosity values that are appropriate for the sample being evaluated. If the contribution of coagulation to the hemostatic process is to be assessed, then the sample must be carefully recalcified to overcome the anticoagulant (105, 106). The whole process is captured using a high-resolution objective lens with a high-speed photodetector or high-sensitivity camera (usually confocal or widefield/fluorescence). Altering channel geometrics can help study platelet aggregation/thrombus formation in conditions recapitulating pathological vessel geometries, stenotic vessels, and vessel areas where stagnation points and shear gradients may occur (107, 108). Microfluidic platforms have been reviewed in detail elsewhere (109, 110).

Many laboratories have used microfluidic systems to monitor thrombus formation, demonstrate the effects of fluid shear stress and define molecular events involved (110–114). Microfluidic studies have assessed thrombus formation in healthy donors (50, 115) and patients afflicted with von Willebrand disease, hemophilia, or thrombocytopenia (114, 116), and used to tease out points of difference between immobilized ligands. De Witt and colleagues ranked 52 different adhesive surfaces for thrombus formation at arterial and venous shear rates (104) and others have studied thrombus formation in blood from mice with genetically engineered deficiencies in platelet receptors or signaling proteins (50). At this time, the only commonly used clinical device that incorporates an element of shear stress to evaluate platelet function is the platelet function analyser (PFA)-100 or PFA-200 which assess time to occlusion of collagen/epinephrine or collagen/ADP coated cartridges by a sample of citrated whole blood. Values in healthy donor samples for time to occlusion are extremely broad and data are unreliable in samples where the hematocrit or platelet count is low (117).

Taken together, findings have led to the consensus that increasing shear stress promotes binding of platelet GPIbα and/or αIIbβ3 to VWF, promoting activation and platelet aggregation (112, 118–120). Exposure to fluid shear stress or immobilization on a solid support matrix modulates VWF tertiary structure, inducing the molecule to unfold and expose sites within the A1 domain of VWF that directly bind to the GPIbα subunit of GPIb-IX-V. This generates signaling events that trigger platelet aggregation (46, 47). Therefore, the effect of pulsatile flow compared to constant flow on thrombus formation will be an important aspect in future studies, especially in the context of platelet activation in mechanical circulatory support devices, such as left ventricular assist devices (LVADs) and extracorporeal membrane oxygenation (ECMO) circuits (34, 108).

Recent studies have also assessed platelet receptor roles in thrombus growth and stability, with GPVI being a key potential player, through its interaction with fibrin in a growing thrombus (121, 122). Targeting of GPVI, a specific receptor found only on platelets and megakaryocytes, provides a good target for anti-platelet therapy without associated bleeding risks (123, 124). Microfluidic systems are therefore a useful tool to examine new GPVI antagonists on reducing thrombus growth and stability. ACT017, a humanized antibody fragment against GPVI, is an example of one of these targets tested with *in vitro* microfluidic systems and has progressed through Phase 1 trials (121, 125, 126).

### Recapitulating a Blood Vessel in a Microfluidics System

Microfluidic imaging systems of whole blood exposed to shear have also provided insight into the contribution of RBCs to thrombus propagation, especially at venous shear rates (121, 127). RBCs are the most abundant blood cell type and are heavily glycosylated. They circulate through the central lumen of the vessel and serve to marginate platelets away from the lumen center and toward the vessel wall. RBCs are the major contributor to blood viscosity, and hence, to vascular fluid shear stress (128–130), which in turn impacts on platelet activity. Therefore, it is important to consider the contribution of RBCs in the design of all microfluidic imaging experiments, particularly when using RBC-free PRPs, or washed platelets. This is typically partially compensated by altering the shear stress in the microfluidic chamber.

Some efforts have been made to grow endothelial cells in microfluidic channels to evaluate endothelial cell contribution to platelet activation and recruitment for forming thrombi (48). Whilst challenging, the seeding, culturing and maintenance of viable endothelial cells to mimic a blood vessel environment in a microfluidic channel formed with a 3D collagen-based hydrogel has been successfully developed (131, 132). However, imaging in thick non-homogeneous cellular network requires good laser scanning microscopy techniques, i.e., intravital microscopy, that will be covered in greater detail in later sections.

### Real Time Microfluidics Quantitative Imaging of Platelets

Thrombus surface area coverage, height and volume are commonly measured in microfluidic devices, but this often requires the use of fluorescently labeled antibodies or probes with variable affinities and efficiencies of binding to the platelet membrane. This approach is not always well-suited for live imaging quantification as these reagents can potentially interfere with normal platelet processes and receptor function. Additionally, laser microscopy increases the risk of photobleaching which, together with phototoxicity, are highly confounding variables during live measurement (133). Further, volumetric quantification is routinely conducted using total fluorescence intensity, which is ultimately limited by the dynamic range of the photodetectors and prone to signal saturation. Other quantitation mechanisms include generalized scoring of thrombi or using the integrated density of the fluorescence signal per field of view (50, 104, 134). These have allowed robust quantitation and comparisons of patient thrombi formed compared to healthy controls, but are not yet standardized approaches, meaning comparison of data across microfluidic systems and between laboratories is not always straightforward (135). Furthermore, most approaches require setting signal thresholds, which can introduce the potential for operator bias and impact on quantitation in real-time.

Although microfluidic systems have advanced the field, these systems do not perform well if coagulation is permitted to proceed. In anticoagulated microfluidic systems the physical properties of thrombi formed do not include the contribution of thrombin activation.

### Imaging Hemostasis and Thrombosis Processes *in vivo*

Whilst imaging of thrombus formation *in vitro* has helped to quantify the contribution of platelet receptors, ligands, and other parameters to thrombus formation, imaging *in vivo* still remains the premier research tool as it permits assessment of thrombus formation in its native microenvironment, which considers contributions from coagulopathy, other blood cells and processes (for example neutrophil extracellular traps) and the endothelium (Table 2). Injury to a blood vessel may be induced using a precisely-guided laser, ligation of a blood vessel, topical application of ferric chloride, or by mechanical or electrolytic injury (150, 151). The selected mode of thrombosis-inducing injury very much depends on the vascular bed being examined and the experimental question being addressed as relative contributions of the surrounding endothelium, transitory leukocytes, and RBCs and the coagulation and complement pathways vary significantly with the mode of injury.

**Table 2:** Imaging techniques and applications for platelet research *in vivo*.

**Imaging method**	**Key points**	**Platelet/cell imaging applications**	**Limitations**	**References**
Confocal scanning	Point scanning microscopy Emitted light selected through a pinhole	Intravital imaging Thrombus formation in mice	Acquisition speed Photobleaching	(136, 137)
1 photon	Excitation with 1 photon laser and illumination of focus and out of focus planes Pinhole distinguishes signal from out of focus plane	Thrombopoiesis (platelet generation)	Limited depth	(80)
2 photon	2 photon laser excitation in focal plane only	Skin Tumor imaging Platelet biogenesis	Limited depth (up to 1 mm) Specialist set up / “in house set ups”	(138)
Spinning disk	Scans sample at multiple points with a CCD camera	Platelet recruitment to injury sites in organs Thrombus formation Platelet-endothelial interactions	Cross-talk between pin holes Limited depth	(139–141)
Multiview SPIM/SIM view	Light-sheet system with switching between 4 pathways	Zebrafish heart development	Limited applications for larger organisms so far Ongoing development for fast 3D scanning	(142, 143)
SHG	2 photons scattered by molecule and emit 1 photon of half excitation wavelength	Collagen/myosin visualization Zebrafish embryos Tumors	Limited to number of structural proteins (unless adding to fluorescence microscopy) Depth limitation	(95, 144, 145)
THG	3 photons scattered by molecule and generate 1 photon of a third of excitation wavelength	Extracellular matrix proteins Tumors	Vessel width limits Excitation power required	(95, 146)
CARS	Non-linear optical process with 3 laser beams (pump, Stokes and probe) Beams interact to generate coherent signal	Tumor imaging/blood flow measurements	New/limited applications so far Complex set up Lacks information on phase	(147–149)

*A non-exhaustive list of imaging techniques used to study platelet generation, function, and roles in thrombus formation in vivo and details of new imaging approaches and current limitations. SPIM, Selective Plane Illumination Microscopy; SIM, Structured Illumination Microscopy; DSLM, Digital Scanned laser Lightsheet fluorescence Microscopy; SHG, Second-Harmonic Generation microscopy; THG, Third Harmonic Generation microscopy; CARS, Coherent Anti-Stokes Raman Scattering microscopy*.

The development of rapid (spinning disk) laser scanning confocal microscopes (152) has enabled sufficient speed to capture transient events in flowing blood. Using laser scanning microscopy, there is sufficient depth penetration to image thrombus formation in mice using laser-induced injury models (136, 153). For example, Falati et al. have assessed the roles of platelet, tissue factor and fibrin in the formation of thrombi in mice after laser-induced endothelial injury (136). However, there is still limited depth which laser confocal microscopy can achieve due to high optical scattering (154). Since then, the inclusion of ultrafast laser systems for multiphoton microscope has open up new opportunities to reach from several hundred micrometers up to 1 millimeter in depth, which expands the hemostasis research questions that can be asked using blood vessels of animals *in vivo* (155). Indeed, the use of the term “intravital microscopy” is now synonymous with the use of multiphoton microscopes across biology.

### Intravital Microscopy Systems

Intravital microscopy systems are now routine to study disease models because of the ability to capture cellular activities in its microenvironment. These instruments use an ultrafast laser system that achieves a reduction of light scattering in tissue and therefore increases depth of imaging. In addition, video rate intravital microscopy offers real-time monitoring so as to record rapid and dynamic events for accurate quantification of events at sub-cellular size scale (156, 157).

Intravital microscopy for thrombosis studies usually requires injection of fluorescently labeled platelets or fluorophore-conjugated antibodies to target a platelet receptor or protein of interest (150, 151). Mice that have been genetically altered to be deficient in a protein or genetically engineered to express reporter-tagged proteins in a cell-specific manner, such as GFP, YFP, and mCherry also have great utility (150, 158–160). Several reports have now used intravital imaging to investigate megakaryocyte-derived structures entering bone marrow sinusoids (161, 162) and platelet production.

### Hemostasis and Thrombus Formation in Its Natural Microenvironment

The development of mice expressing multiple reporters, such as the colorful “confetti” mice reduces the reliance on antibody labeling of cells. For platelet studies, the R26R-Confetti mice were used to study migrating mechanoscavenging platelets that collect bacteria (74). Studies using transgenic mice expressing LifeAct-GFP have also revealed details of platelet actin cytoskeletal structure and nodules (65, 163). When pairing these fluorescent transgenic mice with an intravital microscope, it becomes possible to delve deep underneath dense tissue and potentially observe megakaryopoiesis and changes in ploidy and proplatelet formation and release into the blood stream (87, 164). Fluorescently-labeled platelets have enabled studies of platelet migration and platelet interactions with other blood cells, in a number of physiology scenarios, including inflammation (139), infection (165), and cancer (166).

Key thrombus formation studies *in vivo* have aimed to define the evolution of a thrombus by examining initial steps of platelet activation, signaling and recruitment, and how different extents of platelet activation can affect the stability of the formed thrombus. Stalker and colleagues visualized platelet recruitment following endothelial damage to a cremaster muscle microcirculation, and identified that platelets formed a thrombus with at least 2 distinct zones. The inner core zone contained tightly packed degranulated platelets (as measured by P-selectin expression), which was co-localized with fibrin (167, 168), and had evidence of active thrombin (167, 169). The outer shell zone consisted of loosely-packed platelets, with reduced P-selectin expression and undetectable levels of fibrin. Other intravital laser-induced thrombosis studies have examined roles for tissue factor, thrombin generation (170), platelet receptors GPIbα (171, 172), GPVI (173, 174), protease-activated receptor 4 (175, 176), P2Y_12_ (177, 178), and αIIbβ3 (153, 179, 180). Roles for plasma proteins, such as VWF and fibrinogen (170) fibronectin (181, 182) vitronectin (183) and neutrophil extracellular traps (165) and signaling molecules (184) in platelet activation and accumulation following damage to the endothelium have been defined using intravital imaging systems (150). Thrombi properties vary with the nature of the blood cellular composition and vascular bed as well as the extent of the induced injury, and therefore both factors will determine the response and level of the associated inflammation. This remains a major consideration in the choice of *in vivo* model and imaging modality.

### Limitations of Imaging in Living Organs

A common challenge in intravital imaging is the maintenance of a comparable extent of injury within an animal and across a series of experiments in different batches of animals. This is dependent on consistent laser power, diameter of the laser beam, and depth of the blood vessel. This is especially difficult in confocal systems that often use dual laser sources (i.e., one for imaging and another to induce injury) that require considerable co-alignment in all 3 planar directions to achieve accurate and consistent laser injury. It is possible to use the laser for imaging to also induce laser injury (157) for example in multiphoton imaging, the high-energy near-infrared and infrared pulsed laser allows one to perform laser ablation at a localized section in tissue at a specified depth. To further extend the imaging depth achievable, longer infrared wavelength lasers for triple photon absorption are available and would be a great advantage to platelet researchers but can be limited to the range of excitable fluorophores (185).

The ideal system would allow consistent imaging of platelet recruitment and thrombus formation with minimal photobleaching at any chosen imaging depth. While there are numerous commercial multiphoton intravital microscopes available, the ability to achieve high speed, signal and depth drive many laboratories to build their own systems, which are adapted to the laboratory specifications and requirements (186). However, subtle differences and non-standard configurations mean that experimental conditions cannot be fully duplicated between laboratories.

While the implementation of fluorophores and fluorescent probes are an established method for visualization *in vivo* and *in vitro*, they face various limitations that can impact on the biological application studied or imaged, including interference with receptor signaling, cytotoxicity, and target specificity (Table 2). Thus, the heavy reliance on these biochemical tools can create significant issues with imaging *in vivo* (187, 188). Label-free intravital imaging offers an exciting option to reduce this issue and will allow imaging of platelets and their structures in their physiological environment. A label-free imaging approach will also reduce or remove phototoxicity and photobleaching complications and allow imaging of true dynamic events leading to platelet activation and thrombus formation.

Other potential intravital imaging techniques using multiphoton effects include second and third harmonic generation (SHG, THG) microscopy and Raman scattering (Coherent Anti-stokes Raman scattering; CARS). Many of these modalities have been established in other cell biology systems, and could be applied to intravital mouse thrombosis models (Table 2). SHG microscopy is a non-linear imaging technique, where light scattered over non-centrosymmetric molecules (including the extracellular matrix protein collagen) produces a photon at half the incident wavelength (95). THG microscopy involves non-linear light scattering originating from polarization of an excited volume, including at water-lipid/water-protein interfaces. Thus this approach is relevant to the imaging of molecular events at platelet and cell membranes (146). Raman/CARS microscopy detects signal from inelastic photon scattering upon interaction with matter (95), and would have utility in measuring thrombus volume.

An additional task that all high resolution imaging approaches bring is in the handling and processing of extremely large data files, the necessity to improve contrast and resolution, remove out of focus signal and correct for animal movement (e.g., breathing) that uses image registration (156). In addition to motion, images can be enhanced by going through image deconvolution processes (189, 190). For traditional deconvolution (except for blind deconvolution), it is necessary to first obtain an image of the ideal point spread function of the imaging system. Once the ideal point spread function is determined (191), one can then identify a suitable deconvolution mask to sharpen the images. A mismatch of the ideal point spread function of the system will introduce unnecessary image defects in deconvolved images (192). Upon imaging, it is crucial for imaging specialists to use image registration and deconvolution to improve final images and remove artifacts prior to quantification, in order to reduce errors (193).

### Beyond Fluorescence Imaging: Quantitative Imaging Without Fluorescence Labeling *in vitro*

Reflectance Interference Contrast Microscopy (RICM) (46, 72) is one of the first non-label quantitative imaging approaches that uses interference to examine how platelets interact with an immobilized substrate. Although, this approach is sensitive to several nanometers above the coverslip glass, it is limited to measure signals from a small thickness (~100 nanometers) of a single platelet and cannot be used to quantify volumetric information of platelet aggregates or thrombus.

**Figure 3:**
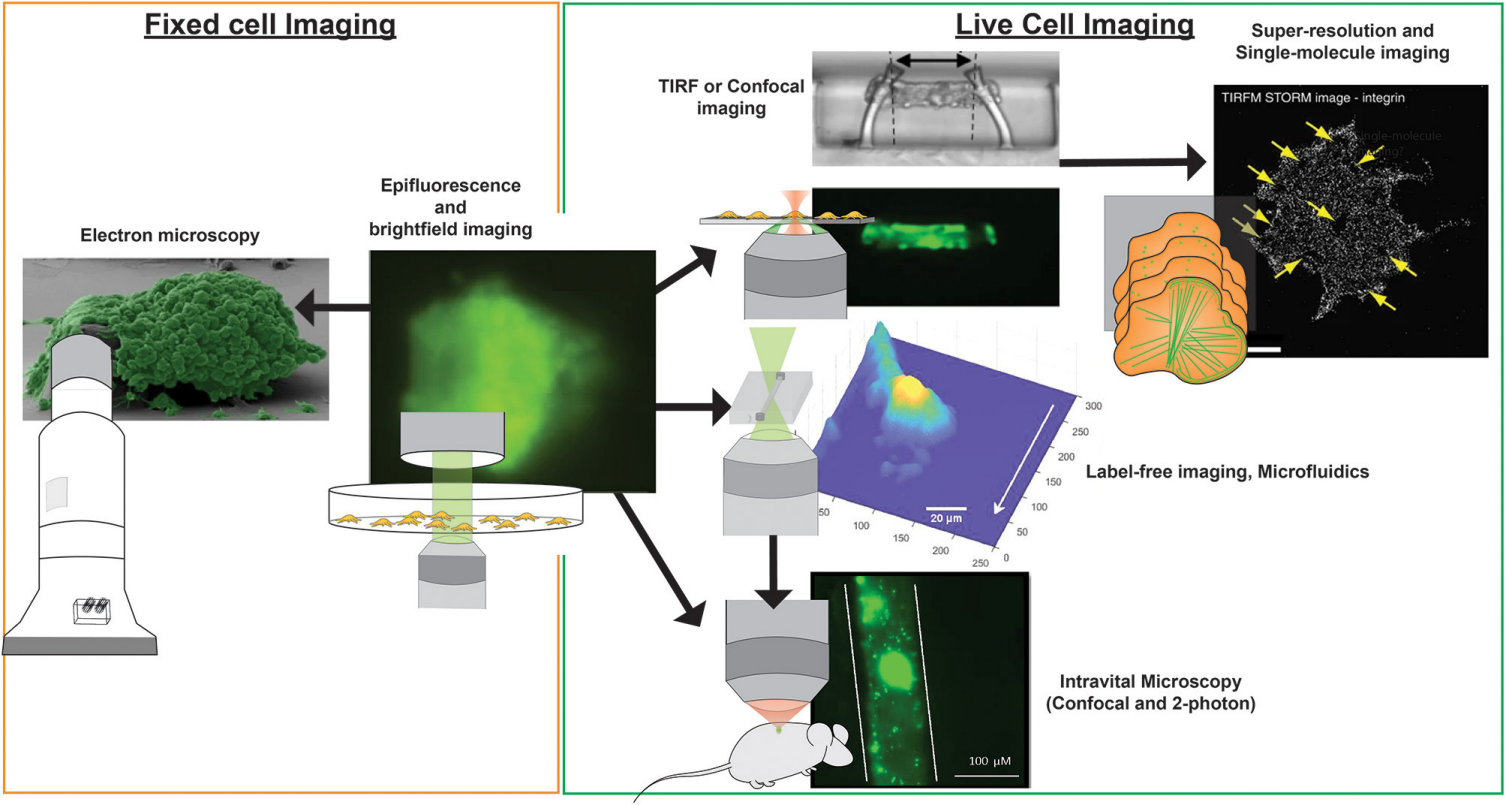
Imaging modalities for visualizing platelets. Multiple imaging modalities can be used for platelet imaging depending on the process to be imaged and imaging environment. Epifluorescence and bright-field imaging are most commonly used for general assessment of thrombus size and the biochemical composition of platelets (195). Electron microscopy allows resolving fine physical structures of single or an aggregate of platelets but is limited to fixed samples (195). Where imaging of functional platelets is required, *in vitro* imaging using TIRF or confocal microscopy could reveal dynamic events of single platelet activity and thrombosis (196), with the option of employing super-resolution and single-molecule imaging techniques for nanometer resolution of fluorescently-tagged biomolecules (65). To recapitulate more physiological conditions, the use of microfluidics and label-free microscopy can provide physiological flow conditions and reduce the risk of phototoxicity incurred by photobleaching, respectively. Finally, *in vivo* platelet imaging has been realized by confocal and 2-photon microscopy, the latter which provides greater tissue penetration and less phototoxicity, but with a higher equipment cost (197). Microscopy images were obtained from https://doi.org/10.1038/s41467-019-09150-9, https://doi.org/10.1038/s41467-019-10067-6, https://doi.org/10.1038/ncomms8254, and https://doi.org/10.1371/journal.pone.0071447, under the Creative Commons license (CC BY 4.0, https://creativecommons.org/licenses/by/4.0/). Images were cropped and figure letters were removed for clarity.

Current standardized microfluidic imaging systems with label-free imaging approaches exploits the refractive index of platelets as its endogenous label. Since there is no nucleus in a platelet, the refractive index of platelets is likely to be stable, providing an opportunity to capture high amounts of quantitative data. Quantitative phase microscopy (QPM) provides measurements of cell depth by monitoring changes in refractive index, which shifts the phase of the incident light wave (53). QPM not only allows non-invasive and label-free imaging of cells, it eliminates the risk of photo-bleaching and reduces optical distortion of samples (54). QPM has been implemented to quantify the volume, mass, and density of platelet aggregates and thrombi formed on collagen-coated microfluidic channels in the presence or absence of tissue factor when exposed to venous shear rates (73). Digital holographic microscopy (DHM), a form of holographic QPM, has been applied to imaging blood samples, and quantify platelet aggregates formed at low (100 s^−1^) shear (194). More recently, DHM was used to quantitatively measure changes in volume of platelet aggregates over time when exposed to different shear rates (Figure 3). A stability index was developed by monitoring the reduction in thrombi volume after the established thrombi field was exposed to elevated (7,000 s^−1^ and 12,000 s^−1^) shear rates using physiological buffered solution (55). The use of QPM with microfluidic systems permits acquisition of accurate values for thrombus height, area and volume without the requirement of fluorescence labeling and the potential to provide a new means of assessing platelet function in clinical samples.

Due to the ease and simplicity of sample handling, QPM techniques can aid predictive models of thrombus formation, contraction, and stability across a range of shear rates and are ideal modalities for development for point-of-care devices to assess platelet function and thrombosis and bleeding risk in at risk patients. These imaging approaches can address research questions targeting mechanisms involved in the regulation of thrombus size, for example the respective roles of metalloproteinases (34, 198) or tetraspanins (199, 200) in modulating thrombus size and stability. However, as QPM techniques rely on phase information in transmitted light, they can often be limited by strongly scattering media. For instance, RBCs are strong scattering agents, akin to tiny polymer lenses, and obscure the visualization of platelets during thrombus formation.

### Integrating High Speed Imaging Into Microfluidic Systems

There is a wealth of molecular tools, platelet-reactive surfaces, microfluidic devices and imaging modalities that sit within research spaces, each approach with specific strengths and weaknesses. Ideally the acquisition of data will be performed under agreed standardized experimental conditions, permitting comparison and integration of findings into current models of thrombus formation under flow.

### *In vitro* Imaging Cytometry

Conventional flow cytometry requires a narrow stream of fluorescently labeled cells in suspension to enter a single weakly focused laser beam. This allows direct single point excitation of fluorescence from each cell passing through the focused laser beam. This approach is devoid of any spatial information with regards to the cell that has been detected and devoid of any morphological information for the detected cell.

Imaging flow cytometry (IFC) aims to incorporate high speed imaging into a flow cytometry system either through a high speed camera system or improved laser scanning methods (201). Although progress in the field of IFC has achieved imaging speed of several hundred imaging frames per second (202), the resolvable image resolutions and imaging depth of these IFC systems can detect platelet/cell aggregates (203, 204) and changes at the platelet membrane (205), but still cannot resolve sub-platelet structures owing to trade-offs between sensitivity, speed, and resolution of the sensor (206).

Currently there are no standardized methods available in the clinical hematology laboratory to evaluate platelet function under vascular fluid shear conditions. Hence, an imaging flow cytometry system that can evaluate platelet function in a standardized way in samples from people with platelet counts below 100 × 10^9^/L would be an asset to clinical hematology. Real time assessment by point-of-care/clinical lab-based imaging flow cytometry devices using microfluidics could help address these issues. For example, information on platelet function in acquired thrombocytopenia at initial presentation and then after therapy could inform on platelet quality particularly if platelet count has not been normalized. Further, monitoring platelet function and thrombotic risk could help guide clinical decisions on patient-specific antiplatelet/anticoagulant therapy to help reduce thrombotic events.

### *In vivo* Imaging Cytometry

The concept of *in vivo* IFC systems was developed to count, characterize, and image biological cells flowing in a living organism (in this case a mouse) at different time points, thus providing longitudinal information of biological events. The *in vivo* flow cytometer was first used to quantify the circulation lifetime of different tumor cells and monitor apoptotic cells in circulation (166, 207, 208) with single cell sensitivity. However, existing *in vivo* IFC approaches are limited by technological challenges that restrict 2D regions of interest to superficial layers of tissue, preventing the experimental interrogation of cellular and molecular events in major blood vessels. Application of laser scanning technology and laser beam shaping, have circumvented this limitation to achieve single cell analysis *in vivo*. For instance, a recent study used multiphoton microscopy techniques to expand the field of *in vivo* imaging tools and visualize calcium fluxes of 10,000 neurons over millimeter ranges (209).

### More Than One Imaging Microscope: Multimodal Imaging

Until now, almost all platelet imaging has been conducted exclusively using a single type of microscope system i.e., spinning disk confocal and structured illumination microscopy. While it is convenient to adopt existing imaging protocols for platelet imaging, there are inherent challenges in imaging platelets due to the complex nature of the platelet-platelet aggregation process. Label-free imaging modalities such as photoacoustic imaging (210) and optical coherence tomography (211) can be combined with an existing multiphoton microscopy system to achieve both structural and fluorescence imaging (212).

## Concluding Remarks

Many brilliant advances in imaging techniques for analyses using *in vivo* and *in vitro* approaches have helped build substantial understanding of roles of receptors, molecular signaling, and the relative contributions of RBCs, leukocytes and platelets to thrombosis and hemostasis as well as other vascular processes. These research-based approaches have been critical for exploration of new means to develop and evaluate novel therapeutics that ultimately improve patient hematology healthcare. The next stage will be to bring one or more of these imaging modalities into the clinical space in an appropriate format and with a well-characterized and standardized ability to evaluate and quantify platelet function in patients at risk of thrombosis, or with unexplained bleeding, or a low platelet count where assessment of platelet function is precluded from using standard hematology laboratory approaches. It is hoped that these tools will also be useful to assess anticoagulant and antiplatelet effectiveness and for stratifying patients who are at risk of thrombosis or bleeding.

## Author Contributions

All authors listed have made a substantial, direct and intellectual contribution to the work, and approved it for publication.

### Conflict of Interest

The authors declare that the research was conducted in the absence of any commercial or financial relationships that could be construed as a potential conflict of interest.
